# Body mass index change trajectories and gastric cancer risk: Effect modification by sex, age, smoking, and menopausal status in Korean adults aged ≥40 years

**DOI:** 10.1371/journal.pone.0350626

**Published:** 2026-05-29

**Authors:** Su Youn Nam, Junwoo Jo

**Affiliations:** 1 Department of Internal Medicine, Kyungpook National University Hospital, Daegu, South Korea; 2 Integrated Research and Treatment Center, School of Medicine, Kyungpook National University, Daegu, South Korea; 3 Department of Statistics, Kyungpook National University, Daegu, Korea; International University of Health and Welfare, School of Medicine, JAPAN

## Abstract

This study examined the association between body mass index (BMI) and gastric cancer risk and specifically evaluated effect modification by sex, age, smoking, and menopausal status. A nationwide cohort of cancer-free Korean adults aged ≥40 years who underwent standardized health examinations in 2009 and 2013 was followed through 2017. BMI was classified into five categories (<18.5, 18.5–22.9, 23–24.9, 25–29.9, and ≥30 kg/m^2^), and BMI changes were grouped into 25 trajectories. Adjusted hazard ratios (aHRs) and 95% confidence intervals (CIs) for gastric cancer were estimated using Cox proportional hazards models, with formal interaction testing for key modifiers. Among 2,800,588 participants (52.8% women), 14,662 incident gastric cancers were identified. Persistent obesity I was consistently associated with an increased gastric cancer risk in total population and most subgroups, including both men and women, both smoking groups, postmenopausal women, and individuals aged < 60 years. In contrast, modest BMI gain from normal to overweight was associated with a reduced risk of gastric cancer in men (aHR 0.90; 95% CI: 0.82–1.00), smokers (aHR 0.88; 95% CI 0.79–0.99), and individuals aged < 60 years (aHR 0.87; 95% CI 0.77–0.98). Persistent obesity II was associated with an increased risk in never smokers. Both underweight and persistent obesity I were associated with an increased risk in those aged < 60 years. BMI change from obesity I to overweight reduced gastric cancer risk (aHR 0.54; 95% CI 0.32–0.92) in premenopausal women, whereas persistent obesity I and BMI gain (underweight to normal) were associated with increased gastric cancer risk in postmenopausal women; however, these findings were exploratory. In conclusion, BMI changes are associated with gastric cancer risk, with clear effect modification by sex, age, and smoking status.

## Introduction

Gastric cancer remains a major global health burden, ranking as the fifth leading cause of cancer-related death globally, with an estimated 660,175 deaths according to Global Cancer Incidence, Mortality and Prevalence (GLOBOCAN) 2022 [1]. Although the age-standardized incidence of gastric cancer has gradually declined in Korea, it continues to represent a substantial public health concern [2,3]. Obesity is a modifiable factor related to gastric cancer [4,5]. However, most prior studies have focused on body mass index (BMI) measured at a single time point, which does not capture dynamic changes in adiposity over time. Emerging evidence suggests that longitudinal changes in obesity indices may provide additional insight into cancer risk beyond baseline BMI alone [6–8]. Recent studies investigated BMI change in relation to cancer risk, primarily focusing on transitions within obese or normal-weight populations [9–11]. In a Korean study, persistent high BMI was associated with increased risk of several digestive cancers, particularly among men [10]. Despite these advances, evidence regarding BMI change and gastric cancer risk remains limited and inconsistent [10,12]. Importantly, little is known about whether the association between BMI change and gastric cancer risk differs across key population subgroups. Effect modification by sex, age, smoking status, and menopausal status is biologically plausible given known differences in hormonal milieu, metabolic regulation, and inflammatory pathways, yet has not been systematically investigated in relation to BMI change and gastric cancer risk. Therefore, using a large nationwide Korean cohort, we systematically examined the association between BMI change and gastric cancer risk. A key novelty of this study is the comprehensive evaluation of effect modification by sex, age group, smoking status, and menopausal status, allowing us to identify subgroup-specific patterns that may be masked in overall analyses.

## Methods

### Data extraction

In this large cohort study, we used data from the Korean National Health Insurance Service System (NHISS), which included the National General Health Examination (NGHE) and the national cancer screening program. The dataset was accessed for research purposes on [06/01/2022] and [10/03/2022]. The database includes demographic data (age, biological sex registered in NHISS, economic status, chronic disease, medication, habits, family history, use of lipid lowering drugs, physical activity) and laboratory examination such as body mass index (BMI, weight/height^2^ [kg/m^2^]), blood pressure, and 12 hours fasting glucose levels (mg/dL). Age is defined as the age at the time when persons underwent NGHE. Economic status was assessed using household income, categorized into 20 quantiles, with 1 representing the lowest income group and 20 the highest income group. Smoking status was categorized as never, past, and current smoker. Alcohol drinking frequency was classified as none, 1/week, 2–3/week, 4–5/week, and ≥6/week. Moderate physical activity referred to “walking or exercising and feeling mild dyspnea for more than 30 min per day.” Women’s factors included menopausal status, breast feeding duration, parity, and estrogen replacement therapy.

### Handling of missing data

Missing data for hypertension and diabetes mellitus in self-reported questionnaires was replaced using blood pressure, and fasting glucose extracted from general health examination. Among missing data of hypertension, systolic blood pressure ≥ 140mHg or diastolic blood pressure ≥ 90mHg was defined as hypertension. Among missing data of diabetes mellitus, fasting glucose ≥ 126 mg/dL was defined as presence of diabetes. Missing data for ischemic heart disease (I20-I25) and cerebrovascular disease (I60-I69) was replaced using T20 disease code within 1 year. Other categorical missing data were put as blank or unknown. Missing data for continuous variables such as age, body mass index, and lipid was put as blank.

### Baseline enrollment and follow-up

**Fig 1:**
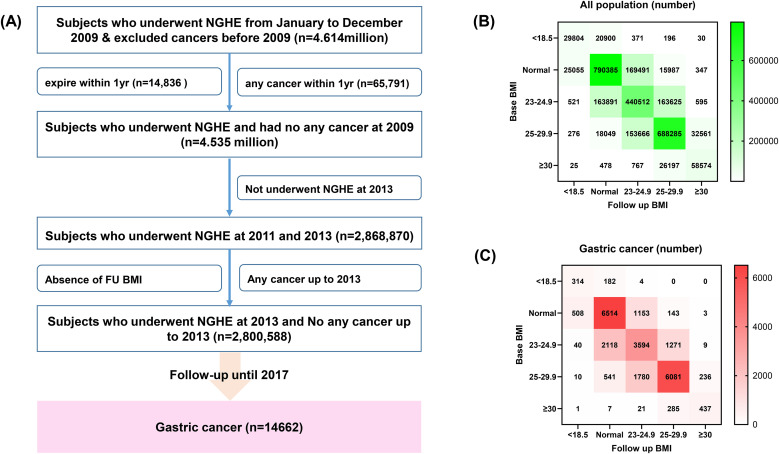
Study overview. **(A)** Enrolments of participants. **(B)** Distribution of all population according to BMI change category. **(C)** Distribution of gastric cancer according to BMI change category. BMI, body mass index; FU, follow-up; NGHE, National Health Examination.

NHISS provided raw data after excluding persons with gastrointestinal cancer from 2004 to 2007. Participants who underwent NGHE and cancer screening in 2009 and underwent NGHE in 2013 were included. Among subjects who underwent both NGHE and National Cancer Screening in 2009 (4.614million), patients with any C-codes within 12 months of baseline enrollment and those who died within 12 months of enrollment were excluded. Individuals who did not undergo NGHE in 2013 and those who had been diagnosed with any cancer before the second measurement of BMI (**Fig 1A**). We extracted all cancer codes (C codes) of the International Classification of Disease (10th revision [ICD 10]) from January 2014 to December 2017. Primary outcome is gastric cancer (C16). Cancer diagnoses in the NHIS require formal cancer registration and physician certification to receive copayment reduction, supporting high diagnostic validity. Patients’ informed consent was exempted because the NHISS provided raw data to researchers after deleting of personally identifiable information and this study did not affect the individual’s disease course. The Institutional Review Board of the Kyungpook National University Chilgok Hospital approved this study (KNUCH 2017-12-022).

### Statistical analysis

Because this study was based on a fixed nationwide cohort, the sample size was determined by the number of eligible individuals. We conducted a theoretical sample size estimation (Supplementary Method in S1 File). The estimated minimum required sample size was 1,627,944, accounting for the 25 BMI trajectory categories and the distribution of participants across BMI groups. The final analytic cohort of 2,800,588 individuals exceeded this threshold, supporting adequate statistical power for the primary analyses. Summary statistics for the variables are provided as numbers (percentages) for categorical variables and means (standard deviations) for continuous variables. We calculated person-years from baseline age to the first date of diagnosis of cancer, death, or end of the study (December 31, 2017). BMI at baseline (2009) and follow-up (2013) was classified into 5 groups according to Asian pacific guideline; [13] <18.5 (underweight), 18.5–22.9 (normal), 23–24.9 (overweight), 25–29.9 (obesity I), ≥ 30 kg/m^2^ (obesity II). Then, BMI change was classified into 25 groups.

Continuous BMI change did not demonstrate a statistically meaningful association with gastric cancer risk in preliminary analyses using linear and restricted cubic spline models; sparse observations in extreme BMI transition cells precluded stable knot placement, consistent with challenges reported in prior studies using continuous BMI change metrics in cohort data with skewed exposure distributions. Therefore, we categorized BMI change into 25 trajectories based on transitions between standard BMI categories to better capture distinct patterns of weight gain, loss, and stability over time. This categorization approach aligns with methods employed in comparable studies examining longitudinal BMI change and cancer risk, where clinically meaningful BMI thresholds provide a basis for interpretable, policy-relevant groupings. This trajectory-based classification was chosen for clinical interpretability rather than etiologic precision, and readers should be aware that some associations may be sensitive to the boundaries of the categories used.

Cancer risk according to BMI change was estimated with hazard ratios (HRs) and 95% confidence intervals (CIs) using the Cox proportional regression analysis. We set the persistent normal BMI as the reference in the main analysis. We set the persistent obesity I as the reference in subgroup analysis among baseline obese persons. The risk by covariates was also assessed using the Cox-regression analysis. We conducted an age- and sex-adjusted model and a multivariate analysis adjusted for significant covariates, including age, sex, economic status, hypertension, diabetes, cerebrovascular disease, heart disease, smoking status, alcohol intake, physical activity, family history of cancer, and the use of lipid lowering drugs. Smoking and menopausal status were treated as baseline covariates assessed at the time of initial health examination and were not updated during follow-up; the implications of this analytical choice, including the potential for non-differential misclassification and its expected direction of bias, are discussed in the Limitations section. Among the final eligible population, missing rate for economic status (1.7%), physical activity (1.6%), and drinking status (1.4%) was higher than 1% and the missing rate of other variables less than 1%. Therefore, we deleted missing data list-wise in the adjusted analysis. We conducted interaction analysis (joint test) between the potential important cofactors (sex, age, smoking, and menopausal status) and BMI change in cancer risk. And subgroup analysis was performed by sex, age (baseline age < 60 and age ≥ 60 years), and smoking (never and ever smokers) and menopausal status (premenopausal and postmenopausal). Analysis of women was further adjusted for women’s factors. All analyses were conducted using SAS software (version 9.4; SAS Institute, Cary, NC, USA). All statistical tests were two-sided, and values of P < 0.05 were considered statistically significant.

## Results

### Baseline demographic and laboratory findings

After the exclusion of participants with any cancer within 1 year and those who died within 1 year, 4.614 million persons were included at baseline. A total of 2.869 million individuals underwent follow-up NGHE in 2013. After further excluding any cancer up to 2013, 2.8 million individuals (47.6% men; mean age of 54.0 years) were eligible and followed up until 2017 (**Fig 1A**). Baseline demographic characteristics (**Table 1**) and women factors (S1 Table in S1 File) according to baseline BMI and the distribution of the overall population and gastric cancer according to BMI change (**Fig 1B**, **1C**) were provided. The total person year was 23,411,813 years and 14662 gastric cancer patients had newly detected cancers during follow-up periods.

**Table 1:** Baseline characteristics by baseline BMI group.

	Baseline BMI, kg/m^2^				
	<18.5	Normal	23-24.9	25-29.9	≥30
Number	51301	1001265	769144	892837	86041
Men, no (%)	21257 (41.4)	415819 (41.5)	388712 (50.5)	472275 (52.9)	34309 (39.9)
Age, mean (SD)	54.0 (11.7)	53.1 (10.0)	54.3 (9.7)	54.8 (9.7)	54.4 (9.9)
Income, mean (SD)	11.1 (5.9)	11.4 (5.9)	11.8 (5.9)	11.9 (5.9)	11.4 (5.8)
Body mass index, mean (SD)	17.6 (0.8)	21.3 (1.1)	23.9 (0.6)	26.7 (1.3)	31.8 (8.5)
Fasting glucose, mean (SD)	93.6 (21.1)	95.6 (20.7)	98.8 (22.5)	101.8 (24.2)	106.5 (28.0)
Triglyceride, mean (SD)	93.8 (62.6)	112.8 (75.2)	137.9 (90.0)	158.5 (101.5)	170.3 (105.6)
Hypertension, no (%)	5674 (11.1)	159507 (16.0)	184780 (24.0)	294798 (33.0)	40240 (46.8)
Heart disease, no (%)	769 (1.5)	17102 (1.7)	17787 (2.3)	25950 (2.9)	3134 (3.6)
Cerebrovascular disease, no (%)	378 (0.7)	7690 (0.8)	7572 (1.0)	10081 (1.1)	1020 (1.2)
Diabetes mellitus, no (%)	1938 (3.8)	52868 (5.3)	57809 (7.5)	87708 (9.8)	12627 (14.7)
Alcohol consumption, no (%)					
None	33212 (65.7)	599787 (60.8)	430325 (56.7)	493442 (56.0)	53713 (63.3)
1/week	11300 (22.3)	268402 (27.2)	224468 (29.6)	259096 (29.4)	21406 (25.2)
2-3/week	3417 (6.8)	76333 (7.7)	70451 (9.3)	89008 (10.1)	6933 (8.2)
4-5/week	1333 (2.6)	24320 (2.5)	20188 (2.7)	24694 (2.8)	1783 (2.1)
≥ 6/week	1317 (2.6)	18407 (1.9)	13246 (1.8)	14635 (1.7)	1073 (1.3)
Smoking status, no (%)					
Never	34566 (67.8)	693913 (69.8)	496217 (65.0)	562333 (63.5)	60712 (71.1)
Past	4900 (9.6)	125389 (12.6)	134106 (17.6)	169165 (19.1)	12159 (14.2)
Current	11510 (22.6)	174663 (17.6)	133081 (17.4)	154782 (17.5)	12555 (14.7)
Moderate physical activity, day/week, mean(SD)	1.0 (1.7)	1.2 (1.8)	1.3 (1.9)	1.3 (1.9)	1.1 (1.8)
Family history of gastric cancer	4409 (10.0)	88844 (10.5)	67901 (10.5)	77726 (10.3)	7286 (9.8)
Lipid lowering drug, no (%)	585 (1.1)	21077 (2.1)	24902 (3.2)	37613 (4.2)	5051 (5.9)

BMI, body mass index; SD, standard deviation.

### Impact of BMI change on gastric cancer risk

Cancer risk was estimated only for BMI change groups with more than five incident gastric cancer cases (**Fig 1C**). In the age- and sex-adjusted model, BMI gain from normal to obesity I and persistent obesity I was associated with an increased risk of gastric cancer, whereas a decreased risk of gastric cancer was observed among individuals with persistent obesity II.

However, this apparently discordant finding for persistent obesity II in the unadjusted model should be interpreted with caution, as the number of participants in this trajectory cell was small, resulting in a wide confidence interval and potentially unstable estimate; this association was no longer apparent in the fully adjusted model. Findings based on sparse data should therefore be regarded as hypothesis-generating rather than confirmatory. Throughout the analyses, results for BMI trajectory groups with small event counts or wide confidence intervals — particularly for extreme BMI trajectories such as persistent underweight, persistent obesity II, and rare transition groups — should be interpreted with particular caution given the inherent instability of such estimates. In the adjusted analysis, persistent obesity I was associated with an increased risk of gastric cancer (aHR 1.12; 95% CI 1.06–1.17) compared with persistent normal BMI (**Table 2**). BMI loss among individuals with obesity was also associated with an increased gastric cancer risk compared with persistent normal BMI.

**Table 2:** Gastric cancer risk according to BMI change.

				Unadjusted analysis		Adjusted analysis			
Base BMI	Follow-up BMI	All population	Case, no (%)	HR (95% CI)	P-value	HR (95% CI) *	P-value	HR (95% CI) *	P-value
**<18.5**	**<18.5**	29804	171 (0.60)	1.22 (1.04-1.42)	0.01	1.00 (0.86-1.17)	0.99	0.95 (0.80-1.13)	0.58
**<18.5**	**Normal**	20900	115 (0.57)	1.17 (0.97-1.40)	0.1	1.10 (0.91-1.33)	0.31	1.06 (0.86-1.30)	0.57
**Normal**	**<18.5**	25055	161 (0.67)	1.35 (1.16-1.59)	<0.001	1.01 (0.86-1.19)	0.88	0.99 (0.83-1.18)	0.92
**Normal**	**Normal**	790385	3736 (0.49)	1		1		1	
**Normal**	**23-24.9**	169491	717 (0.44)	0.89 (0.82-0.97)	0.005	0.91 (0.84-0.98)	0.01	0.93 (0.85-1.02)	0.11
**Normal**	**25-29.9**	15987	94 (0.61)	1.24 (1.01-1.52)	0.04	1.23 (1.00-1.51)	0.04	1.15 (0.91-1.45)	0.24
**23-24.9**	**Normal**	163891	944 (0.60)	1.21 (1.13-1.30)	<.0001	1.04 (0.97-1.12)	0.24	1.07 (0.99-1.16)	0.09
**23-24.9**	**23-24.9**	440512	2309 (0.54)	1.11 (1.05-1.17)	<.0001	1.01 (0.96-1.06)	0.76	1.02 (0.96-1.08)	0.62
**23-24.9**	**25-29.9**	163625	843 (0.53)	1.09 (1.01-1.17)	0.03	1.06 (0.98-1.14)	0.14	1.04 (0.95-1.13)	0.421
**25-29.9**	**Normal**	18049	117 (0.68)	1.35 (1.13-1.63)	0.001	1.09 (0.91-1.31)	0.35	1.09 (0.89-1.33)	0.41
**25-29.9**	**23-24.9**	153666	885 (0.60)	1.22 (1.13-1.31)	<.0001	1.03 (0.95-1.10)	0.49	1.03 (0.95-1.12)	0.42
**25-29.9**	**25-29.9**	688285	3964 (0.60)	1.22 (1.17-1.28)	<.0001	1.10 (1.06-1.15)	<.0001	1.12 (1.06-1.17)	<.0001
**25-29.9**	**≥30**	32561	160 (0.51)	1.04 (0.89-1.22)	0.62	1.13 (0.96-1.32)	0.13	1.17 (0.99-1.39)	0.069
**≥30**	**25-29.9**	26197	143 (0.57)	1.15 (0.97-1.36)	0.10	1.14 (1.01-1.29)	0.03	1.23 (1.03-1.47)	0.021
**≥30**	**≥30**	58574	286 (0.51)	1.04 (0.92-1.17)	0.57	0.37 (0.36-0.39)	<.0001	1.13 (0.99-1.29)	0.072

*Adjusted for age and sex.

**Adjusted for age, sex, income, hypertension, heart disease, stroke, diabetes mellitus, smoking status, drinking status, physical activity, lipid lowering drug, family history of cancer. BMI, body mass index; CI, confidence interval; HR, hazard ratio.

### Effect modification by sex and smoking status

**Fig 2:**
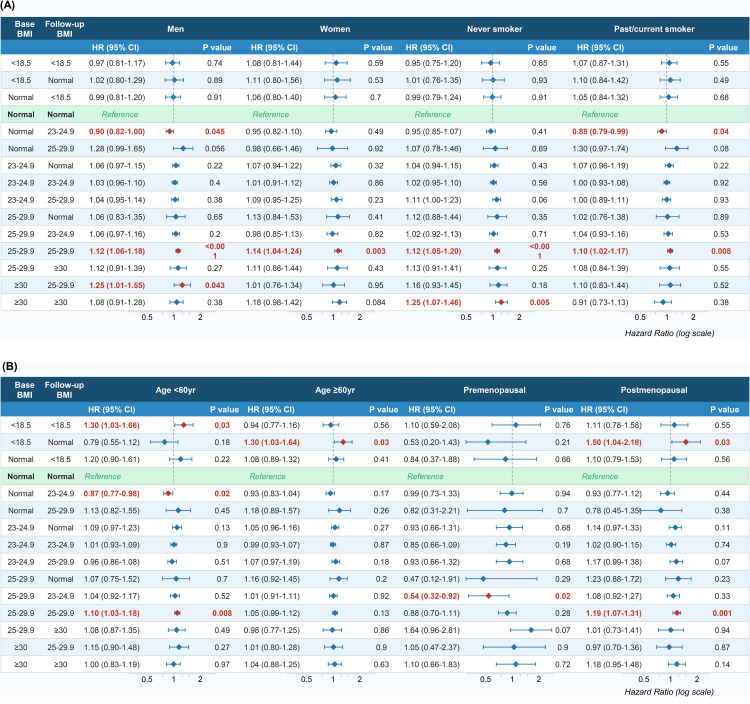
Association between BMI change and gastric cancer risk: Sub-group analysis. **(A)** Subgroup analysis by sex and smoking status. **(B)** Subgroup analysis by age group and menopausal status. Adjusted for age, sex, economic status, hypertension, diabetes, cerebrovascular disease, heart disease, smoking status, alcohol intake, physical activity, family history of cancer, and the use of lipid lowering drugs. ^†^Adjusted for age, income, hypertension, heart disease, stroke, DM, smoking status, drinking status, physical activity, lipid lowering drug, breast feeding, parity, use of oral contraceptives, family history of cancer. aHR, adjusted hazard ratio; BMI, body mass index; CI, confidence interval; FU, follow-up. Reference: persistent normal BMI (Normal → Normal). *p < 0.05, **p < 0.01, ***p < 0.001.

Formal interaction testing demonstrated statistically significant interactions between BMI change and sex (P for interaction <0.0001), as well as between BMI change and smoking status (P for interaction <0.0001) (S2 Table in S1 File). Stratified results are presented to describe effect modification supported by these interaction tests. Persistent obesity I was associated with an increased risk of gastric cancer in both men and women. Modest BMI gain among normal weight (normal to overweight) was associated with a reduced risk of gastric cancer (aHR 0.90; 95% CI 0.82–1.00; p = 0.045), but marked BMI gain (normal to obesity I) is borderline associated with an increased risk of gastric cancer (aHR 1.28; 95% CI 0.99–1.65; p = 0.056) in men. (**Table 3 and Fig 2A**).

**Table 3:** Gastric cancer risk according to BMI change: Subgroup analysis by sex and smoking status.

			Sex						Smoking				
			Men			Women			Never smoker			Past or current smoker	
Base BMI	Follow-up BMI	Case	HR (95% CI)*	P value	Case	HR (95% CI)*	P value	Case	HR (95% CI)*	P value	Case	HR (95% CI)*	P value
<18.5	<18.5	120	0.97 (0.81-1.17)	0.74	51	1.08 (0.81-1.44)	0.59	73	0.95 (0.75-1.20)	0.65	97	1.07 (0.87-1.31)	0.55
<18.5	Normal	76	1.02 (0.80-1.29)	0.89	38	1.11 (0.80-1.56)	0.53	51	1.01 (0.76-1.35)	0.93	63	1.10 (0.84-1.42)	0.49
Normal	<18.5	108	0.99 (0.81-1.20)	0.91	52	1.06 (0.80-1.40)	0.70	79	0.99 (0.79-1.24)	0.91	81	1.05 (0.84-1.32)	0.68
Normal	Normal	2538	1		1194	1		1916	1		1795	1	
Normal	23-24.9	493	0.90 (0.82-1.00)	0.045	223	0.95 (0.82-1.10)	0.49	360	0.95 (0.85-1.07)	0.41	354	0.88 (0.79-0.99)	0.04
Normal	25-29.9	66	1.28 (0.99-1.65)	0.056	28	0.98 (0.66-1.46)	0.92	41	1.07 (0.78-1.46)	0.69	50	1.30 (0.97-1.74)	0.08
23-24.9	Normal	646	1.06 (0.97-1.15)	0.22	296	1.07 (0.94-1.22)	0.32	498	1.04 (0.94-1.15)	0.43	441	1.07 (0.96-1.19)	0.22
23-24.9	23-24.9	1689	1.03 (0.96-1.10)	0.40	617	1.01 (0.91-1.12)	0.86	1148	1.02 (0.95-1.10)	0.56	1143	1.00 (0.93-1.08)	0.92
23-24.9	25-29.9	589	1.04 (0.95-1.14)	0.38	254	1.09 (0.95-1.25)	0.23	434	1.11 (1.00-1.23)	0.06	406	1.00 (0.89-1.11)	0.93
25-29.9	Normal	70	1.06 (0.83-1.35)	0.65	47	1.13 (0.84-1.53)	0.41	70	1.12 (0.88-1.44)	0.35	44	1.02 (0.76-1.38)	0.89
25-29.9	23-24.9	633	1.06 (0.97-1.16)	0.20	250	0.98 (0.85-1.13)	0.82	461	1.02 (0.92-1.13)	0.71	421	1.04 (0.93-1.16)	0.53
25-29.9	25-29.9	2818	1.12 (1.06-1.18)	<0.001	1145	1.14 (1.04-1.24)	0.003	2011	1.12 (1.05-1.20)	<0.001	1931	1.10 (1.02-1.17)	0.008
25-29.9	≥30	93	1.12 (0.91-1.39)	0.27	67	1.11 (0.86-1.44)	0.43	92	1.13 (0.91-1.41)	0.25	65	1.08 (0.84-1.39)	0.55
≥30	25-29.9	90	1.25 (1.01-1.55)	0.043	53	1.01 (0.76-1.34)	0.95	88	1.16 (0.93-1.45)	0.18	55	1.10 (0.83-1.44)	0.52
≥30	≥30	150	1.08 (0.91-1.28)	0.38	135	1.18 (0.98-1.42)	0.084	193	1.25 (1.07-1.46)	0.005	90	0.91 (0.73-1.13)	0.38

*Adjusted for age, sex, income, hypertension, heart disease, stroke, diabetes mellitus, smoking status, drinking status, physical activity, lipid lowering drug, family history of cancer. BMI, body mass index; CI, confidence interval; HR, hazard ratio.

With respect to smoking status, persistent obesity I (aHR 1.12; 95% CI 1.05–1.20) and II (aHR 1.25; 95% CI 1.07–1.46) were associated with an increased gastric cancer risk among never smokers, whereas persistent obesity I (aHR 1.10; 95% CI 1.02–1.17) was associated with an increased gastric cancer risk and modest weight gain (normal to overweight) was associated with a reduced gastric cancer risk in ever smokers (**Table 3 and Fig 2A**).

### Effect modification by age and menopausal status

A significant interaction was identified between age group (<60 vs. ≥ 60 years) and BMI category (P < 0.0001). Among individuals younger than 60 years, persistent underweight and persistent obesity I were associated with an increased gastric cancer risk but modest BMI gain from normal to overweight was associated with a reduced gastric cancer risk. In contrast, BMI gain from underweight to normal was associated with an increased gastric cancer risk among those aged 60 years or older (S3 Table in S1 File **and Fig 2B**).

In contrast, no statistically significant interaction was identified between menopausal status and BMI change in joint tests. Although stratified analyses by menopausal status were conducted based on prior literature suggesting possible cancer stage [14], our findings should be interpreted as exploratory. Among premenopausal women, BMI loss from obesity I to overweight was associated with a reduced gastric cancer risk (aHR 0.54; 95% CI 0.32–0.92), whereas persistent obesity I (aHR 1.19; 95% CI 1.07–1.31) and BMI change from underweight to normal change from underweight to normal (aHR 1.50; 95% CI 1.04–2.18) **were** associated with an increased gastric cancer risk in postmenopausal women (S3 Table in S1 File; **Fig 2B**).

## Discussion

In this large cohort study, persistent obesity was consistently associated with an increased risk of gastric cancer risk in overall population, men, and women. The association had a difference by smoking and age. Persistent underweight and persistent obesity I were associated with increased gastric cancer risk, but modest BMI gain among normal BMI reduced risk in individuals aged <60 years. BMI gain among individuals with underweight was associated with an increased gastric cancer risk in individuals older than 60 years.

BMI gain from normal to obesity I and persistent obesity I were associated with an increased gastric cancer risk in the age- and sex-adjusted model, and this association for persistent obesity I remained significant after full adjustment. These findings suggest that sustained obesity, rather than transient weight change, may confer a stable increase in gastric cancer risk, potentially reflecting prolonged exposure to obesity-related metabolic and inflammatory pathways. In sex-specific analysis, our findings that persistent obesity I was associated with an increased gastric cancer risk in both men and women, was similar to those in a previous study (S4 Table in S1 File). Modest BMI gain from normal to overweight was associated with a reduced gastric cancer risk in men. In non-cardiac gastric cancer prevalent regions such as Korea, [15] the association between baseline BMI and gastric cancer risk has been reported in L, inverted J, or U shapes [16–18].

To the best of our knowledge, this is the first study to investigate effect modification by smoking, age, and menopausal status in the association between gastric cancers and BMI changes. In never smokers, persistent obesity I and II were associated with an increased risk, suggesting that obesity-related metabolic dysfunction, inflammation, and insulin resistance may play a dominant role in gastric carcinogenesis. In contrast, among ever smokers, persistent obesity I increased gastric cancer risk, but modest BMI gain from normal to overweight was linked to a reduced risk.

This apparent protective association should be interpreted cautiously and may reflect the complex metabolic consequences of smoking and smoking cessation rather than a direct benefit of adiposity. Smoking increases basal metabolic rate, suppresses appetite, and promotes systemic inflammation and oxidative stress, while smoking cessation is often accompanied by modest weight gain and partial metabolic recovery. Thus, modest weight gain in ever-smokers may partially reflect partial reversal of smoking-induced catabolic and nutritional depletion, potentially attenuating inflammatory pathways. Furthermore, nicotine suppresses ghrelin and alters leptin signaling, and smoking cessation-associated weight gain may restore adipokine balance toward a more metabolically favorable profile. Even if one small study reported that the BMI change had no association with colorectal cancer incidence [19], smoking-specific modification of BMI change–gastric cancer relationships has not been previously reported and warrants further investigation.

Age also modified the association, with persistent underweight and obesity I increasing gastric cancer risk and BMI gain from normal to overweight reducing risk in those younger than 60 years, whereas BMI gain from underweight to normal increased risk in those aged 60 years or older. These age-specific patterns likely reflect differences in metabolic reserve, inflammatory tone, and hormonal responsivity: moderate weight gain in younger individuals may indicate improved nutritional status, whereas the same gain in older adults may exacerbate underlying vulnerabilities such as sarcopenia-related inflammation or atrophic gastritis. Although stratified analyses suggested differences in the association between BMI change and gastric cancer risk by menopausal status, interaction testing did not support statistically significant effect modification. Therefore, these findings should be considered exploratory. In premenopausal women, BMI loss from obesity I to overweight was associated with a reduced risk, possibly reflecting metabolic improvement following weight reduction. In postmenopausal women, persistent obesity I and BMI gain from underweight to normal were associated with increased gastric cancer risk. Notably, both the reduced risk associated with BMI loss from obesity I to overweight in premenopausal women (aHR 0.54; 95% CI 0.32–0.92) and the increased risk associated with BMI gain from underweight to normal in postmenopausal women (aHR 1.50; 95% CI 1.04–2.18) are based on small event counts with wide confidence intervals, and should therefore be regarded as hypothesis-generating rather than confirmatory. These exploratory findings suggest that menopausal status may merit consideration in future studies examining the relationship between BMI changes and gastric cancer risk.

The increased gastric cancer risk observed with weight gain from underweight to normal weight in older adults or postmenopausal women requires careful interpretation. Rather than indicating a healthy return to normal weight, late-life weight gain in underweight individuals may reflect underlying metabolic dysregulation, sarcopenia-to-fat transition, or age-related chronic inflammatory states. Prior studies have reported higher gastric cancer risk at both underweight and obese extremes [16–17], supporting the biological plausibility that weight gain in underweight older individuals may not confer metabolic benefit but indicate increased vulnerability to carcinogenesis. Estrogen may exert pleiotropic anti-inflammatory and immune-modulating effects on the gastric mucosa, partly through suppression of pro-inflammatory cytokines (e.g., interleukin-6, tumor necrosis factor-α) and modulation of gastric acid secretion, whereas androgen-related visceral adiposity and sex-specific adipokine profiles may contribute to differential risk by sex and age. Furthermore, sex-specific differences in host immune responses to *H. pylori* infection — partly mediated by estrogen-driven modulation of mucosal immunity and adipokine signaling — may further amplify or attenuate obesity-related carcinogenic pathways in a sex-dependent manner. In premenopausal women, BMI loss from obesity I to overweight may reduce adipose-derived aromatase activity and normalize estrogen levels within a physiologically favorable range, potentially attenuating obesity-driven inflammatory carcinogenesis. After menopause, declining estrogen, increasing central adiposity, insulin resistance, and pro-inflammatory signaling may amplify obesity-related carcinogenic pathways, supporting a modifying hormonal milieu rather than BMI alone [20]. In the context of high *H. pylori* prevalence in Korea, postmenopausal loss of estrogen-mediated immune protection may further enhance susceptibility to *H. pylori*-driven mucosal injury, potentially synergizing with obesity-related metabolic dysfunction to elevate gastric cancer risk.

An important consideration in interpreting BMI change trajectories is the potential for reverse causality, whereby unintentional weight loss due to preclinical disease may precede gastric cancer diagnosis. In this study, we excluded cancer or death within 1 years after baseline enrollment and further excluded cancers before the second BMI measurement to avoid the possibility of reverse causation. Our findings do not support reverse causality as a dominant explanation, as weight loss was not associated with increased gastric cancer risk, whereas persistent obesity consistently showed higher risk across most analyses. The absence of uniformly increased risk among weight-loss trajectories does not eliminate reverse causation but suggests that sustained obesity, rather than short-term weight change, may be a more stable marker of gastric cancer risk. Moreover, modest BMI gain from normal weight to overweight was associated with lower risk in specific subgroups, and BMI loss from obesity I to overweight reduced risk in premenopausal women, patterns more consistent with metabolic improvement than disease-related wasting. Reverse causation cannot be fully excluded, as weight loss may sometimes reflect underlying or subclinical disease rather than intentional lifestyle modification. The recently proposed concept of clinical obesity emphasizes that BMI does not fully capture functional abnormalities of adipose tissue or obesity-related health impairment [21]. Although BMI change shares these limitations, repeated BMI measurements better characterize cumulative exposure and weight dynamics than single-time-point BMI, providing insight into transitions toward obesity-related metabolic dysfunction and cancer risk.

The heterogeneous associations between BMI trajectories and gastric cancer risk are biologically plausible. Adipose tissue promotes insulin resistance, hyperinsulinemia, IGF-1 signaling, and pro-inflammatory adipokine secretion, all of which may facilitate gastric epithelial transformation [22]. Importantly, in East Asian populations where Helicobacter pylori infection is highly prevalent and non-cardia gastric cancer predominates, the interaction between *H. pylori*-induced chronic inflammation and obesity-related metabolic dysfunction may play a critical role in carcinogenesis. *H. pylori* infection induces persistent gastric mucosal inflammation characterized by increased production of pro-inflammatory cytokines such as interleukin-6 and tumor necrosis factor-α [23], while obesity further amplifies systemic inflammatory signaling and insulin resistance [22]. These overlapping inflammatory and metabolic pathways may synergistically enhance epithelial damage, genomic instability, and tumor initiation. East Asian studies incorporating *H. pylori* have reported a U-shaped association between BMI and gastric cancer risk [17], consistent with our findings of elevated risk at both extremes. This non-linear association may reflect distinct biological mechanisms: underweight status may be associated with malnutrition-related immune impairment and reduced host defense against *H. pylori*, whereas obesity may promote a pro-inflammatory and hyperinsulinemic milieu that facilitates *H. pylori*-related carcinogenesis. Furthermore, metabolic alterations associated with obesity, including insulin resistance and altered adipokine signaling, may modify host responses to *H. pylori* infection and influence gastric mucosal susceptibility. The apparent protective association of modest weight gain in men, ever-smokers, and younger individuals — as discussed above — likely reflects improved metabolic reserve or smoking cessation-related recovery rather than a direct adiposity benefit, a pattern also noted in Asian cohorts [18]. These counterintuitive protective findings are intriguing but should be explicitly regarded as exploratory; given the multiple comparisons performed, they may reflect chance findings and require replication in independent cohorts before any clinical conclusions can be drawn. Sex-specific differences in BMI- and glucose-gastric cancer associations further underscore the importance of metabolic and hormonal context [16]. These associations should be interpreted as hypothesis-generating, warranting confirmation in studies with more detailed metabolic, infectious, and behavioral data.

Our findings suggest that longitudinal changes in adiposity — rather than baseline BMI alone — may be relevant to gastric cancer risk, with effects that differ by age, sex, smoking status, and menopausal status. These findings do not support uniform weight-management strategies; instead, they highlight the importance of life-course timing, health status, and behavioral context. Future studies incorporating intentionality of weight change, body composition, screening patterns, and biological markers are needed before translation into preventive strategies.

This study has remarkable strengths. First, we explored the impact of BMI change on gastric cancer risk in a large nationwide cohort. Second, we systematically explored effect modification by sex, age, smoking, and menopausal status in the association between BMI change and risk of gastric cancers. To the best of our knowledge, this is the first study to assess age-, smoking-, and menopause-related heterogeneity in the association between BMI change and gastric cancer risk. Third, we used high-quality NHISS data, including directly measured BMI and detailed information on many covariates, thereby enhancing the validity and robustness of our findings.

This study also has several limitations. First, the follow-up duration was relatively short; however, the large sample size and person-years partially mitigate this concern. Second, behavioral changes during follow-up (smoking and menopausal status) were not considered, leading to potential misclassification and attenuating true interaction effects. This misclassification is expected to be non-differential with respect to the outcome, as smoking or menopausal status changes during follow-up are unlikely to be systematically related to subsequent cancer diagnosis. Non-differential misclassification of effect modifiers typically biases interaction estimates toward the null, meaning that the observed subgroup-specific associations are likely conservative and the true effect modification may be underestimated.

Although major comorbidities and incomes were adjusted, residual confounding cannot be entirely excluded. Third, multiple BMI change categories and subgroup analyses increase the likelihood of type I error. However, the primary findings — in particular, the consistently elevated risk with persistent obesity I across subgroups and the reduced risk with modest BMI gain — were directionally concordant across strata, reducing the probability that they represent isolated false-positive findings. In contrast, associations observed in only one or a few strata, particularly those based on small event counts or unsupported by significant interaction testing, should be considered hypothesis-generating rather than confirmatory, and interpreted with caution to avoid overinterpretation of marginal associations. Subgroup findings by menopausal status, not supported by significant interaction testing, should be interpreted as exploratory. In addition, as detailed in the Methods, the categorical BMI change approach, while chosen for clinical interpretability, may have obscured finer dose–response relationships.

Fourth, *H. pylori* and several important risk factors, including dietary factors, medication use, family history, and detailed alcohol intake, were unavailable, raising the possibility of residual confounding. To quantify this uncertainty, we calculated E-values for the primary association (persistent obesity I vs. persistent normal BMI; aHR 1.12, 95% CI 1.06–1.17). The E-value was 1.49 for the point estimate and 1.31 for the lower confidence limit, indicating that an unmeasured confounder would need risk ratio associations of at least 1.49 on both pathways to fully explain the findings. Both values were derived using the formula E-value = aHR + √[aHR × (aHR − 1)], applied to the point estimate (aHR 1.12; E-value 1.49) and the lower confidence limit (aHR 1.06; E-value 1.31), allowing readers to verify the calculations directly. These E-values indicate that only moderate unmeasured confounding would be needed to explain the observed associations, and residual confounding therefore cannot be excluded. Accordingly, the observed associations should be interpreted cautiously and should not be regarded as definitively causal, particularly given the observational design and the unavailability of key variables such as *H. pylori* status and dietary factors. Major known confounders were adjusted for in the analysis, partially mitigating this concern, but the possibility that unmeasured or inadequately measured factors contributed to the findings remains. The very low prevalence of metabolic/bariatric surgery in Korea during the study period (227 cases in 2009) [24] is unlikely to have materially influenced BMI trajectory estimates. Fifth, inclusion of participants who completed two consecutive health examinations may have introduced a “healthy participant” effect. Although prior studies have identified sociodemographic rather than BMI-specific predictors of gastric cancer screening participation in Korea [25], differential screening rates by BMI trajectory cannot be formally excluded. The direction and magnitude of any resulting detection bias remain uncertain. If individuals with obesity are less likely to attend screening, obesity-associated risk may be underestimated; conversely, if they attend more frequently, detection bias could inflate risk estimates. We are unable to formally quantify or adjust for this potential bias within the current dataset, and this uncertainty should be considered when interpreting the observed associations. Sixth, competing risks from non–gastric cancer deaths were not explicitly modeled. Because our primary objective was etiologic inference rather than absolute risk estimation, cause-specific Cox models were considered appropriate; Fine–Gray competing risk models would be indicated had the goal been to estimate absolute cumulative incidence. Seventh, BMI does not capture fat distribution, lean mass, or intentionality of weight change, and associations may therefore reflect heterogeneous metabolic processes. Cardia gastric cancer is more strongly associated with obesity, while non-cardia gastric cancer is primarily driven by *H. pylori* infection, dietary salt intake, and smoking, with a more variable BMI association. As cancer subtype and stage information were unavailable, subtype-specific BMI trajectory effects could not be examined, potentially obscuring heterogeneous associations across histological subtypes [18]. As some differing aetiologies of cardia and non-cardia gastric cancer suggest that the overall BMI trajectory associations reported here may represent a mixture of subtype-specific effects that differ in direction or magnitude. Specifically, if BMI-related carcinogenic pathways predominantly affect cardia gastric cancer — which is less prevalent in Korean cohorts — the associations observed in this study may underestimate true effects in the cardia subtype and reflect a composite estimate dominated by non-cardia cancer biology. BMI trajectories were defined using Asian-Pacific cutoffs, which are more appropriate for Korean adults; sensitivity analyses using WHO cutoffs were not performed, potentially limiting comparability with Western studies. Finally, as a single nationwide Korean cohort, external validation was not performed; therefore, findings should be regarded as hypothesis-generating and require replication in non-Asian population.

In conclusion, persistent high BMI was associated with an increased risk of gastric cancer, and the impact of BMI change differed according to sex, age, smoking, and menopausal status. While sustained obesity generally increased risk, modest BMI gain from normal to overweight was associated with a reduced risk in men, ever-smokers, and individuals younger than 60 years, whereas weight gain from underweight increased risk in older adults and postmenopausal women. Our results highlight the importance of considering individual-level characteristics — including sex, age, smoking history, and menopausal status — when interpreting the health implications of BMI change, as associations are not uniform across populations. These findings are hypothesis-generating and require replication before informing clinical or public health recommendations.

## Supporting information

S1 File Supplementary tables. (DOCX)
