# Supplementary material for: Body mass index change trajectories and gastric cancer risk: Effect modification by sex, age, smoking, and menopausal status in Korean adults aged ≥40 years
Source: PLoS One. 2026 May 29;21(5):e0350626. doi: 10.1371/journal.pone.0350626 (PMC13221031; doi:10.1371/journal.pone.0350626)
Supplement: S1 File — (DOCX) [file pone.0350626.s001.docx]

**Supplementary Method**

**Sample size estimation**

Because this study was based on a fixed nationwide cohort, the sample size was determined by the number of eligible individuals rather than by an a priori calculation. However, we conducted a theoretical sample size estimation for the primary comparison to assess whether the available cohort size would provide sufficient statistical power. We assumed that persistent high BMI would increase gastric cancer risk compared with persistently normal BMI, that transitioning from obesity to normal weight would reduce gastric cancer risk compared with persistently obese individuals, and that additional BMI gain among obese individuals would further elevate gastric cancer risk. Using a two-sided α error of 0.05 and 80% power, the required sample size for comparing two independent groups was calculated based on an expected incidence of 0.6% in Group 1 and 0.5% in Group 2, yielding an estimated 171,724 participants. In our study, BMI change patterns were classified into 25 categories. Based on findings from our previous research, approximately 3% of participants were distributed in the lowest and highest BMI categories. Grouping the middle three BMI categories produced nine combination groups (3 × 3). Therefore, the minimum required sample size was calculated as: 171,724 × 9 + 171,724 × 16 × 0.03 (3%)= 1,627,944.

**S1 Table. Baseline women factors by baseline BMI group**

|  | **Baseline BMI** |  |  |  |  |
| --- | --- | --- | --- | --- | --- |
|  | **<18.5** | **Normal** | **23-24.9** | **25-29.9** | **≥30** |
| **Menstruation status, no (%)** |  |  |  |  |  |
| Premenopausal | 12724 (47.3) | 229799 (43.8) | 109473 (31.7) | 98045 (25.4) | 12717 (26.6) |
| Hysterectomy state | 1143 (4.3) | 31296 (6.0) | 25025 (7.2) | 30346 (7.9) | 4033 (8.4) |
| Post-menopause | 13046 (48.5) | 263320 (50.2) | 211420 (61.1) | 258379 (66.8) | 31087 (65.0) |
| **Estrogen replacement therapy, no (%)** |  |  |  |  |  |
| Never | 10454 (80.9) | 201593 (77.3) | 163884 (78.2) | 208274 (81.3) | 26282 (85.4) |
| < 2yrs | 1190 (9.2) | 28284 (10.8) | 21731 (10.4) | 22858 (8.9) | 2079 (6.8) |
| ≥2yrs to <5yrs | 473 (3.7) | 12468 (4.8) | 9044 (4.3) | 8425 (3.3) | 661 (2.2) |
| ≥5yrs | 337 (2.6) | 9048 (3.5) | 7156 (3.4) | 6762 (2.6) | 534 (1.7) |
| Unknown | 469 (3.6) | 9524 (3.7) | 7676 (3.7) | 9760 (3.8) | 1224 (4.0) |
| **Parity, no (%)** |  |  |  |  |  |
| 1 | 3680 (13.7) | 55170 (10.5) | 27424 (7.9) | 26216 (6.8) | 3354 (7.0) |
| ≥2 | 21269 (79.1) | 448799 (85.6) | 308489 (89.1) | 349980 (90.5) | 43031 (89.9) |
| Never | 1957 (7.3) | 20542 (3.9) | 10160 (2.9) | 10603 (2.7) | 1464 (3.1) |
| **Breast feeding duration, no (%)** |  |  |  |  |  |
| <6 months | 5389 (20.2) | 87689 (16.8) | 39198 (11.4) | 33226 (8.6) | 3738 (7.9) |
| ≥ 6 to <12 months | 5526 (20.7) | 123895 (23.8) | 74361 (21.6) | 73021 (19.0) | 8203 (17.3) |
| ≥12 months | 10915 (41.0) | 244063 (46.8) | 198934 (57.8) | 247941 (64.5) | 31534 (66.3) |
| Never | 4823 (18.1) | 65888 (12.6) | 31679 (9.2) | 30401 (7.9) | 4087 (8.6) |
| **Use of oral contraceptive pill, no (%)** |  |  |  |  |  |
| Never | 22512 (84.0) | 426823 (81.6) | 275060 (79.8) | 303771 (78.8) | 37385 (78.4) |
| <1yr | 2148 (8.0) | 47824 (9.1) | 32894 (9.5) | 37471 (9.7) | 4637 (9.7) |
| ≥1yr | 965 (3.6) | 23348 (4.5) | 19104 (5.5) | 24054 (6.2) | 3199 (6.7) |
| Unknown | 1172 (4.4) | 24960 (4.8) | 17827 (5.2) | 20202 (5.2) | 2443 (5.1) |

BMI, body mass index

**S2 Table. Interaction analysis (Joint test)**

|  | **By sex** |  | | | **By age** |  | | |
| --- | --- | --- | --- | --- | --- | --- | --- | --- |
| **Dependent variable** | **Effect** | **DF** | **Wald Chi-Square** | **P value** | **Effect** | **DF** | **Wald Chi-Square** | **P value** |
| Gastric cancer | SEX_TYPE*BMI13_cat | 4 | 67.4247 | <.0001 | age_cat_60*BMI13_cat | 4 | 60.2962 | <.0001 |
|  | **By smoking** |  |  |  | **By menopausal status** |  |  |  |
| Gastric cancer | SMK*BMI13_cat | 8 | 100.2048 | <.0001 | QC_MNS_YN*BMI13_cat | 8 | 7.5536 | 0.47 |

age_cat_60, age group (<60 years or ≥60years); BMI13_cat, BMI change (persistent underweight, normal, overweight, obesity I, obesity II); DF, degree of freedom; QC_MNS_YN, menstruation status (premenopausal, hysterectomy, post-menopausal); SMK, smoking status (never, past, current)

**S3 Table. Gastric cancer risk according to BMI change: Subgroup analysis by age and menopausal status**

|  |  |  | **By age** |  |  |  |  |  | **By menopausal status** | |  |  |  | |  |
| --- | --- | --- | --- | --- | --- | --- | --- | --- | --- | --- | --- | --- | --- | --- | --- |
|  |  |  | **Age<60yr** |  |  | **Age≥60yr** |  |  | **Premenopausal** |  |  | **Postmenopausal** | | |  |
| **Base BMI** | **Follow-up BMI** | **Case** | **HR (95% CI)*** | **P value** | **Case** | **HR (95% CI)*** | **P value** | **Case** | **HR (95% CI)*** | **P value** | **Case** | **HR (95% CI)*** | | **P value** |  |
| <18.5 | <18.5 | 71 | 1.3 (1.03-1.66) | 0.03 | 100 | 0.94 (0.77-1.16) | 0.56 | 11 | 1.10 (0.59-2.08) | 0.76 | 37 | 1.11 (0.78-1.58) | | 0.55 |  |
| <18.5 | Normal | 36 | 0.79 (0.55-1.12) | 0.18 | 79 | 1.30 (1.03-1.64) | 0.03 | 5 | 0.53 (0.20-1.43) | 0.21 | 31 | 1.50 (1.04-2.18) | | 0.03 |  |
| Normal | <18.5 | 49 | 1.2 (0.9-1.61) | 0.22 | 112 | 1.08 (0.89-1.32) | 0.41 | 6 | 0.84 (0.37-1.88) | 0.66 | 42 | 1.1 (0.79-1.53) | | 0.56 |  |
| Normal | Normal | 1687 | 1 |  | 2049 | 1 |  | 288 | 1 |  | 794 | 1 | |  |  |
| Normal | 23-24.9 | 340 | 0.87 (0.77-0.98) | 0.02 | 377 | 0.93 (0.83-1.04) | 0.17 | 52 | 0.99 (0.73-1.33) | 0.94 | 157 | 0.93 (0.77-1.12) | | 0.44 |  |
| Normal | 25-29.9 | 41 | 1.13 (0.82-1.55) | 0.45 | 53 | 1.18 (0.89-1.57) | 0.26 | 4 | 0.82 (0.31-2.21) | 0.70 | 17 | 0.78 (0.45-1.35) | | 0.38 |  |
| 23-24.9 | Normal | 365 | 1.09 (0.97-1.23) | 0.13 | 579 | 1.05 (0.96-1.16) | 0.27 | 39 | 0.93 (0.66-1.31) | 0.68 | 238 | 1.14 (0.97-1.33) | | 0.11 |  |
| 23-24.9 | 23-24.9 | 1051 | 1.01 (0.93-1.09) | 0.90 | 1258 | 0.99 (0.93-1.07) | 0.87 | 85 | 0.85 (0.66-1.09) | 0.19 | 476 | 1.02 (0.90-1.15) | | 0.74 |  |
| 23-24.9 | 25-29.9 | 386 | 0.96 (0.86-1.08) | 0.51 | 457 | 1.07 (0.97-1.19) | 0.18 | 37 | 0.93 (0.66-1.32) | 0.68 | 201 | 1.17 (0.99-1.38) | | 0.07 |  |
| 25-29.9 | Normal | 35 | 1.07 (0.75-1.52) | 0.70 | 82 | 1.16 (0.92-1.45) | 0.20 | 2 | 0.47 (0.12-1.91) | 0.29 | 41 | 1.23 (0.88-1.72) | | 0.23 |  |
| 25-29.9 | 23-24.9 | 355 | 1.04 (0.92-1.17) | 0.52 | 530 | 1.01 (0.91-1.11) | 0.92 | 15 | 0.54 (0.32-0.92) | 0.02 | 222 | 1.08 (0.92-1.27) | | 0.33 |  |
| 25-29.9 | 25-29.9 | 1855 | 1.10 (1.03-1.18) | 0.008 | 2109 | 1.05 (0.99-1.12) | 0.13 | 116 | 0.88 (0.70-1.11) | 0.28 | 962 | 1.19 (1.07-1.31) | | 0.001 |  |
| 25-29.9 | ≥30 | 85 | 1.08 (0.87-1.35) | 0.49 | 75 | 0.98 (0.77-1.25) | 0.86 | 15 | 1.64 (0.96-2.81) | 0.07 | 49 | 1.01 (0.73-1.41) | | 0.94 |  |
| ≥30 | 25-29.9 | 65 | 1.15 (0.90-1.48) | 0.27 | 78 | 1.01 (0.80-1.28) | 0.90 | 6 | 1.05 (0.47-2.37) | 0.90 | 44 | 0.97 (0.70-1.36) | | 0.87 |  |
| ≥30 | ≥30 | 143 | 1.00 (0.83-1.19) | 0.97 | 143 | 1.04 (0.88-1.25) | 0.63 | 16 | 1.10 (0.66-1.83) | 0.72 | 111 | 1.18 (0.95-1.48) | | 0.14 |  |

*Adjusted for sex, income, hypertension, heart disease, stroke, DM, smoking status, drinking status, physical activity, lipid lowering drug, breast feeding, parity, use of oral contraceptives, family history of cancer.

BMI, body mass index; CI, confidence interval; HR, hazard ratio; NA, not applicable.

**S4 Table. Previous cohort studies for obesity index change-cancer association and current study**

| **Cancer site (ICD10)**  **1^st^ author year** | **Design/location** | **size** | **Time Frame for Body Size Change** | **Obesity-Cancer risk: overall, By sex** | **By age group** | **By smoking, menopausal status** |
| --- | --- | --- | --- | --- | --- | --- |
| **Stomach (C16)** |  |  |  |  |  |  |
| Current study | Cohort/Korea | 2.8million | ≥40 years (midlife to elderly)  (median FU: 8.4 year) | **Overall GC risk**  aHR 1.12 (1.06-1.17) in persistent obesity I  aHR 1.23 (1.03-1.47) in obesity II🡪 obesity I  comparing to **persistent normal BMI.**  **By sex: both significant**  Persistent obesity I: aHR 1.12 (men), 1.14 (women)  obesity II🡪 obesity I: aHR 1.25 (men), NS (women)  excessive weight gain (men)  normal to obesity I: aHR 1.28 (0.99-1.68)  overweight to obesity II):, 2.46 (0.92-6.56)  **Among obese persons**,  **BMI reduction**  aHR 0.93 (0.86-1.01) (obesity I 🡪 overweight)  comparing to **persistent obesity I group.** | (reference) persistent normal  **<60yr**  1.30 (1.03-1.66) (persistent low BMI)  0.87 (0.77-0.98) (normal🡪 overweight)  1.10 (1.03-1.18) (persistent obese I)  **≥60yr**  1.30 (1.03-1.64) (low BMI 🡪 normal) | **By smoking** (similar pattern)  **By menopausal**  **(premenopausal)**  0.54 (0.32-0.92) (obesity I🡪 overweight)  1.64 (0.96-2.81) (obesity I🡪 obesity II)  **(postmenopausal)**  1.5 (1.04-2.18) (Underweight 🡪normal)  1.19 (1.07-1.31) (Persistent obese) |
| Tran 2022 | Cohort/Korea | 1.88 million men/  899,912 women | ≥19 years | **Reference (persistent normal)**  **(men)**  Persistent underweight: 0.76 (0.65-0.90)  Underweight-gain : 0.62 (0.65-0.90)  Persistent obesity I: 1.10 (1.03-1.16)  Persistent Obesity II, III : 1.26 (1.09-1.45)  **(women)**  Persistent obesity I: 1.11 (1.00-1.24)  Obesity II, III🡪 moderate loss: 1.28 (1.01-1.63)  **Among obese persons**,  BMI loss or gain : NS in both men and women | NA | NA |
| Sofia Christakoudi 2021 | EPIC cohort | 241,323 | 40-70yr  (8 years) | Weight change: NS | NA | NA |

GC, gastric cancer; NA, not assessed; NS, not significant
